# A Brain Centred View of Psychiatric Comorbidity in Tinnitus: From Otology to Hodology

**DOI:** 10.1155/2014/817852

**Published:** 2014-06-11

**Authors:** Massimo Salviati, Francesco Saverio Bersani, Giuseppe Valeriani, Amedeo Minichino, Roberta Panico, Graziella Francesca Romano, Filippo Mazzei, Valeria Testugini, Giancarlo Altissimi, Giancarlo Cianfrone

**Affiliations:** ^1^Department of Sensory Organs, Sapienza University of Rome, Rome, Italy; ^2^Department of Neurology and Psychiatry, Sapienza University of Rome, Rome, Italy

## Abstract

*Introduction*. Comorbid psychiatric disorders are frequent among patients affected by tinnitus. There are mutual clinical influences between tinnitus and psychiatric disorders, as well as neurobiological relations based on partially overlapping hodological and neuroplastic phenomena. The aim of the present paper is to review the evidence of alterations in brain networks underlying tinnitus physiopathology and to discuss them in light of the current knowledge of the neurobiology of psychiatric disorders. *Methods*. Relevant literature was identified through a search on Medline and PubMed; search terms included tinnitus, brain, plasticity, cortex, network, and pathways. *Results*. Tinnitus phenomenon results from systemic-neurootological triggers followed by neuronal remapping within several auditory and nonauditory pathways. Plastic reorganization and white matter alterations within limbic system, arcuate fasciculus, insula, salience network, dorsolateral prefrontal cortex, auditory pathways, ffrontocortical, and thalamocortical networks are discussed. *Discussion*. Several overlapping brain network alterations do exist between tinnitus and psychiatric disorders. Tinnitus, initially related to a clinicoanatomical approach based on a cortical localizationism, could be better explained by an holistic or associationist approach considering psychic functions and tinnitus as emergent properties of partially overlapping large-scale neural networks.

## 1. Introduction

Comorbid psychiatric disorders are frequent among patients affected by tinnitus [1]. In ancient times, Hippocrates and then Galen remarked the frequent concomitant presentation of tinnitus and depressive symptoms (melancholia), hypothesizing that the effect of black bile (*atra bilis*) on the same organ, the brain, could represent the common etiopathogenetic factor of the two disorders [2]. In the course of history both tinnitus [3] and psychiatric disorders [4] have been considered the expression of pathological alterations of various different organs potentially having mystic or unknown aetiology.

Current medical literature indicates that the association between tinnitus and psychiatric disorders is complex [5]. Those elements underlying the frequent, multiform, and nondeterministic relation between the two classes of disorders will be evidenced in this introduction from epidemiological, clinical, and biological points of view.

Both the classes of disorders are common in the general population, with a prevalence of 15–20% of tinnitus and 27% of psychiatric disorders [6]. From an epidemiological point of view, the prevalence of comorbid psychiatric disorders among tinnitus patients ranges between 14% and 80% [7, 8], with such a large range probably due to the different methodologies of sampling and diagnosis used in the different clinical studies [9]. Two recent studies of our research team found comorbid psychiatric disorders in 48% [10] and 43% [11] of the enrolled tinnitus patients. It is also true, however, that patients suffering from tinnitus-related distress may more frequently seek clinical help and thereby may have a better chance to get enrolled in clinical studies than patients with well compensated tinnitus; for this reason, the prevalence of high psychiatric comorbidity in tinnitus may be only representative of the subpopulation of clinical help seekers.

Although the majority of studies on the topic are focused on comorbid depression, other psychiatric disorders have also been found to be substantially present in tinnitus patients, such as anxiety, obsessive compulsive, mood, conversion, somatoform [12], sleep [13], psychotic [14], cognitive [15], substance use related [16], language [17], sexual [18], personality [18], and eating disorders [19]. In addition, some authors reported that the rate of suicide among tinnitus patients is 10 times higher than among general population [20].

The temporal relation between tinnitus and psychiatric disorders is not linear: psychiatric comorbidities are not simply reactive to tinnitus distress but they can even precede tinnitus onset [6]. It is still not possible to postulate the presence of a psychopathologically determined vulnerability to tinnitus onset but, on the other hand, preliminary studies of our research team on temperament and character provide evidence of a personological predisposition (scarce coping abilities and neurotic prone attitude) for the development of a disabling and distressful perception of tinnitus (i.e., severe tinnitus) [10]. A recent study of Sand et al. [21] on gene variants of glial cell-derived neurotrophic factor (GDNF) and brain-derived neurotrophic factor (BDNF) in tinnitus patients provided interesting links between coping skills and the degree of tinnitus-related distress; BDNF Val66Met gene has been further the object of extensive investigations in sensitivity to stress and adaptation to stress [22] and empirical data support its additional roles in the processing of auditory information [23] and in the tinnitus severity in women [24].

Stressful life events and daily hassles may precede tinnitus onset [25], can contribute to tinnitus physiopathology [26], or may be elicited by decompensated tinnitus [27]. Furthermore, there are mutual clinical influences between psychiatric disorders and tinnitus: tinnitus severity and its impact on quality of life lead to more severe presentations of the concomitant psychopathological disorders, while concomitant psychopathological disorders can strongly worsen the tinnitus-related distress potentially representing the milestones to shift from a compensated to a decompensated tinnitus [28].

The complex circular relationship between psychopathology and tinnitus has strongly stimulated the scientific debate; the major issue underlying the theoretical speculations about this comorbidity is the unobjectifiable nature of the clinical manifestations of the two classes of disorders: both of them are not identifiable through objective diagnostic markers but rather through subjective symptoms resulting from a functional impairment of the same organ, the brain [29].

Disturbances of connectivity and thus of neural dynamics are thought to underlie a number of disease states of the brain, and some evidence suggests that degraded functional performance of brain networks may be the outcome of a process of randomization affecting their nodes and edges [30].

In tinnitus, as well as in psychiatric disorders, neural plasticity, defined as the adaptation of central nervous system (CNS) to altered peripheral input and the compensation of the effects induced by injury or diseases, occurs in all parts of the central nervous system; it represents an allostasis attempt occurring after a deprivation of peripheral input, after an abnormal peripheral input or injury, learning, and adaptation, and even after behavioral training. A large amount of current researches focuses on the concept of maladaptive neural plasticity to explain the physiopathology of tinnitus and psychiatric disturbances [31], identifying the phenomenon leading to multiple pathological conditions that we globally define as “dysfunctional de-contextualizations from sensorial experience fields” (i.e., bodily perceptions, environmental embodiment, and the otherness) [32]. This plastic reorganization causes neuronal or even glial and vascular changes at molecular, cellular, and histological levels [33, 34].

The fundamental processes underlying neural plasticity at molecular levels may be traced to two mechanisms: protein phosphorylation (i.e., a rapid, easily reversible response) and regulation of genes expression (i.e., a more structured process) [33]. Brain reorganization may emerge quickly or slowly and may be permanent or labile, reflecting a shift in the influence of excitatory or inhibitory events in the brain [35]. The changes may involve the synaptic communication between neurons but also the cellular membrane properties [35].

**Figure 1:**
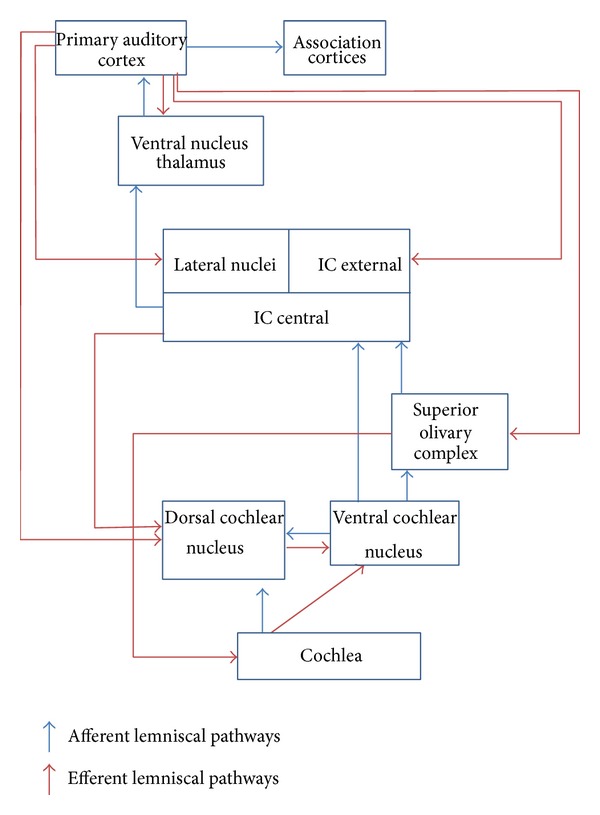
Lemniscal pathways, modified from [34]. Abbreviations: IC = Inferior Colliculus.

**Figure 2:**
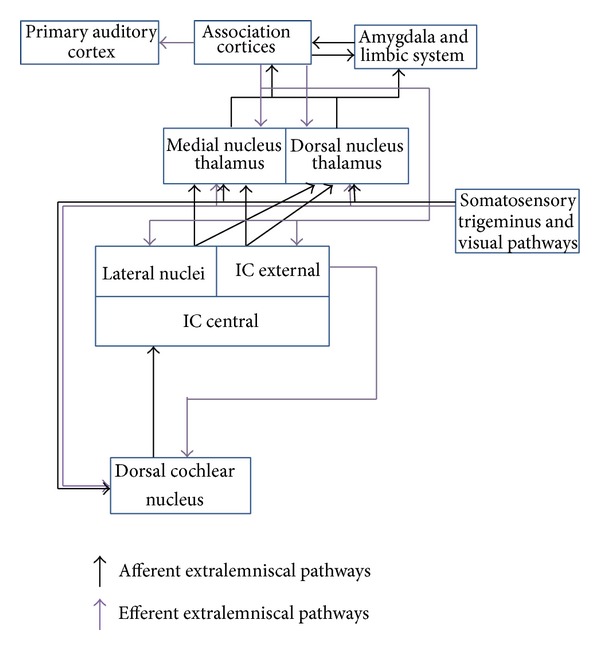
Extralemniscal pathways, modified from [34]. Abbreviations: IC = Inferior Colliculus.

According to the deafferentation-based pathogenetic model of tinnitus, it is possible to individuate two different stages or levels of neuronal plastic reorganizations and network reconfigurations in tinnitus. During the initial response to peripheral input deprivation, neural plasticity induces an allostatic response in the auditory cortex, consisting in a reduced GABAergic inhibition of dormant, glutamate excitatory synapses, and creates new excitatory connections through axonal sprouting and lateral spread of neural activity, resulting in enlarged regions of neural activity [34]. These reorganization processes and new axonal connections contribute to an excess of tonotopical cells representing a very restricted tonotopical area of the cochlea, perceived as tinnitus [34]. It is assumed that this “lateral spread” of these excitatory response areas creates conditions of hyperexcitability in the brain [34]. In the second stage of plastic reorganization the new auditory cortex neuronal restyling is punctuated and limited in function and extension by brain network gating systems; in case of a lack of gating system or in case of the presence of facilitating factors, the neuronal restyling affects several auditory (lemniscal and extralemniscal) and nonauditory pathways, leading to modifications in the location and crossmodal interplay of specific information processes. In fact, there is nowadays evidence that tinnitus phenomenon results from systemic neurootological triggers (Table 1) followed by neuronal remapping within several auditory (Figures 1 and 2) and nonauditory pathways [6].

According to this “remapping” hypothesis of tinnitus, the reorganization process usually begins with a loss of hair cells in the inner ear, a “sensorineural” hearing loss (SNHL) [37–39]. Notably, tinnitus has been reported to occur more frequently in patients with hearing loss, but it occurs even in individuals with normal hearing [40]. When audiological testing is performed at finer intervals and at frequencies above 8 kHz, cases of tinnitus with absolutely no hearing loss become more rare in our hands and in those of other investigators [41]. It is safe to say, therefore, that the great majority of tinnitus cases do involve SNHL, that is, damage to the sensory periphery. Importantly, the reverse is not true; that is, not everyone with SNHL develops tinnitus.

Data on complexity of global interrelation between different brain areas in tinnitus patients derive from resting-state functional magnetic resonance imaging (rfMRI). rfMRI allows to study functional connectivity in the brain by acquiring fMRI data while subjects lie inactive in the MRI scanner and taking advantage of the fact that functionally related brain regions spontaneously coactivate.

In healthy subjects, the identified auditory resting-state network encompasses bilateral primary and associative auditory cortices, insula, prefrontal, sensorimotor, anterior cingulate, and left occipital cortices. On the other hand, in tinnitus patients the identified auditory resting-state network has been found to encompass all previously mentioned areas (excluding the anterior cingulate cortex) and also included the brainstem, thalamus, nucleus accumbens (NAc), isthmus of cingulate gyrus, and right occipital, parietal, and prefrontal cortex (PFC). In addition, chronic tinnitus patients as compared to controls showed increased connectivity in the brainstem, cerebellum, right basal ganglia/NAc, parahippocampal areas, right frontal and parietal areas, left sensorimotor areas and left superior temporal region and decreased connectivity in right primary auditory cortex, left fusiform gyrus, and left frontal and bilateral occipital regions [42].

Utilizing fMRI to study psychopathological dimensions, some authors found that diverse forms of psychopathology are characterized by breakdowns (disconnectivity) in interregional relationships between networked brain regions leading to cognitive, affective, motivational, and social dysfunctions [63].

What results clear from the studies of the last decades is that tinnitus and psychiatric disorders cannot be considered only diseases of specific anatomically defined parts of the brain, but rather disorders resulting from complex wide subtle dysfunctions of multiple CNS regions and networks, leading to the idea of diffuse rather than localized disorders potentially sharing common neurobiological substrates [64].

Given the increasing amount of data evidencing epidemiological, clinical, and neurobiological relations and mutual influences between tinnitus and psychiatric disorders, the aim of the present paper is to review the evidence of alterations in brain networks underlying tinnitus physiopathology and to discuss them in light of the current knowledge of the neurobiology of psychiatric disorders.

## 2. Methods

Relevant literature was identified through a search on Medline and PubMed. Search terms included tinnitus[ti] AND brain AND plasticity OR tinnitus[ti] AND brain AND cortex OR tinnitus[ti] AND brain AND network OR tinnitus[ti] AND brain AND pathways. Through these search terms, 139 papers have been found. Among these, we considered only those studies written in English and conducted on humans (109 papers); reviews, meta-analysis, editorials, and letters were excluded, resulting in a total of 66 papers. Among these, we manually selected only those studies fitting the purpose of the review study and investigating alterations in brain networks through neuroimaging and neurophysiological techniques (Tables 2 and 3). Results have been discussed in the light of the current data available about psychiatric disorders neurobiology.

## 3. Results

An association of tinnitus with changes in the function and structure of auditory pathways has been demonstrated in many studies; however, tinnitus-related activity changes within CNS are not restricted to the auditory pathways [74] but rather they can be conceived as alterations of a network involving both auditory (lemniscal and extralemniscal) and nonauditory structures [75, 76].

Auditory networks can be divided into three streams that convey information “*into,”* “*within,” *and “*beyond” *auditory cortex [77].

The primary auditory cortex, in fact, receives projections of the acoustic radiations from both the medial geniculate nuclei and it represents the final step of lemniscal and extralemniscal ways (“into” pathway).

The information then flows within auditory cortex (“within” pathway) and connects to adjacent areas through U-shaped fibres. The local connections of each auditory area are unique, complex, and characterized by the following properties: (1) a single area typically has reciprocal connections with several others; (2) adjacent areas tend to be more densely interconnected than nonadjacent areas; (3) the densest connections link neurons within a single area; and (4) laminar and sublaminar patterns of connections vary systematically.

Ultimately, information flows “beyond” auditory cortex (“beyond” pathway) toward the auditory-related areas. In particular, from the auditory cortex information moves in four principal directions (1) rostral, (2) caudal, (3) medial, and (4) lateral. The rostrally directed stream has auditory-related targets in temporal pole, ventral, rostral, and medial prefrontal areas, rostral cingulate, parahippocampal areas, and the amygdala, while the caudally directed stream flows from the caudal belt and parabelt regions into temporoparietal junction, posterior parietal and occipital regions (such as secondary visual cortex), caudal and dorsal prefrontal areas, dorsal cingulate, and parahippocampal areas; the rostral and caudal areas of auditory cortex project, therefore, to auditory-related targets that are largely segregated, many of which are located in regions of the brain associated with the ventral and dorsal networks of the extrastriate visual system. The other two “streams” (medial and lateral) flow laterally from the belt and parabelt regions to the superior temporal sulcus and medially into the insula and retroinsular areas within the lateral sulcus [77].

The results of the present review are given in Tables 2 and 3; they will be presented into separate sections focusing on afferent (“into”), intracortical (“within”), and efferent (“beyond”) structures discussing the specific brain networks underlying tinnitus physiopathology.

### 3.1. Tinnitus-Related Brain Structures “into” and “within” Auditory Pathways (Table 2)

Both anatomical and functional alterations of auditory pathways are nuclear findings related to tinnitus perception; the auditory cortex has been found to be reduced in volume [43, 50] and altered in functionality [48, 51–55, 59, 61, 62, 67, 68] in numerous studies and its hyperactivity plays a critical role in tinnitus.

fMRI data showed symmetrical activation in the primary auditory cortex in patients with bilateral tinnitus and homolateral activation towards the side of perceived tinnitus in patients with lateralized tinnitus [45, 49], supporting the idea that tinnitus may be considered as an auditory phantom phenomenon.

Simple phantom sounds like tinnitus are related to an increased neuronal activity within the auditory cortex secondary to the imbalance between excitatory and inhibitory mechanisms or an adjustment of auditory gain mechanisms [78]. One major psychoacoustic finding is that the dominant tinnitus pitch generally falls within the area of hearing loss; this is consistent with the theory of deafferentation as the main trigger of hyperactivation of tonotopic cortex in tinnitus pathogenesis [36]. The side of perception of tinnitus can be linked to the side of the altered structures of the auditory pathways.

Altered auditory inputs may support in tinnitus patients widespread functional reorganization of synaptic connections leading to dysfunctional activity in several subcortical lemniscal structures [36, 49, 53, 60–62, 67, 68, 78, 79] (cochlear nuclei, inferior colliculi (IC), and medial geniculate bodies) and associative auditory cortex [67]; Cochlear nuclei (ventral and dorsal) have been found hyperactive [51, 52, 54, 55, 59, 61, 62], IC has been found reduced in volume [44] and both hyper and hypoactive [45, 46, 49] and Medial geniculate bodies have been found hypoactive in left sided tinnitus patients [49]. The contrasting findings could be explained by the different methodologies of the studies and could be interpreted as the effect of a neuroplastic attempt to gate aberrant signals by saturation [80].

In tinnitus the long-term reorganization of central auditory pathways appears to lead to changes at cortical as well as thalamic level, resulting in structural changes (increase of grey matter density in posterior thalamus associated with significant volume loss in subcallosal area [66]) and altered thalamocortical lemniscal and extralemniscal oscillations [81]. According to this model tinnitus perception is related to an abnormal, spontaneous, and constant gamma band activity (>30 Hz) generated as a consequence of hyperpolarization of specific thalamic nuclei [57]; moreover, it was found that tinnitus perceived loudness is correlated with increased contralateral gamma band activity in the auditory cortex indicating that gamma band activity is a frequent founding in tinnitus patients [53, 58]. Based on magnetoencephalography (MEG) data, the emergence of gamma band activity could be also enabled by the absence of thalamus inhibitory function in the auditory cortex, which in turn is shown by reduced alpha band activity (8–12 Hz) [56]. Direct connections from the thalamic nuclei of the nonlemniscal pathway to the limbic system may explain these components often accompanying tinnitus [34].

The limbic system is a group of interconnected cortical and subcortical structures dedicated to linking visceral states and emotion to cognition and behavior; it has always been considered to be a complex arrangement of transitional structures situated between a visceral “primitive” subcortical brain and a more evolved cortical one. It is affected by a wide range of disorders including neurodevelopmental conditions and neurodegeneration [65]. Limbic structures are also considered a part of extralemniscal auditory pathway [34].

Among the limbic structures, the subgenual anterior cingulate cortex extending into nucleus accumbens-ventral tegmental area is involved in the processing of aversive sounds and unpleasant music as well as tinnitus [82]; it is functionally connected to the amygdala, insula, parahippocampus, orbitofrontal cortex, and ventrolateral PFC and anticorrelated with the dorsal anterior cingulate cortex and precuneus and, as such, the subgenual anterior cingulate cortex could be thought to be important as an emotional component for tinnitus [83]. By comparison of patients with tinnitus with high and low distress, differences in neuronal activity were identified in a network of the anterior cingulate cortex, the anterior insula, and the amygdale; this nonspecific distress network is similarly activated in chronic pain or somatoform disorders [65, 72].

Evidence from neuroimaging studies in patients with tinnitus reports increased connectivity in basal ganglia parahippocampal, right prefrontal, parietal, and sensorimotor areas [42] and hyperactivity in the associative auditory cortices and in the left hippocampus [67]. Hippocampal involvement in tinnitus pathophysiology is also documented by MRI evidence of decreased grey matter volume in this area: this result confirms histopathological findings of hippocampus lesions in patients who experience tinnitus as a symptom of methyltin intoxications [84, 85]. Other relevant findings (fMRI and encephalographic studies) focus on parahippocampal area whose involvement in tinnitus might be related to the establishment of auditory memory for tinnitus [86].

Even if the limbic activation has traditionally been interpreted as a reflection of the emotional reaction of tinnitus patients to the tinnitus sound, limbic and paralimbic structures may play a more extended role than previously proposed. According to a recent paper [30], efferents from structures in the subcallosal area, which includes the nucleus accumbens of the ventral striatum and the ventral medial PFC, are involved in the cancellation of the tinnitus signal at the thalamic level. Although the tinnitus signal may initially be generated in parts of the auditory system, it is the failure of the limbic regions to block this signal that leads to the tinnitus percept becoming chronic [30]. Limbic areas seem to be involved both in chronicization and in decompensation of tinnitus.

Tinnitus distress is related to neural activity in left and right anterior insula according to some authors [58, 72, 76]. The insula is part of auditory pathways and, together with the dorsal anterior cingulate cortex, has also been referred to as the salience network [87]. This network has been implicated in bottom-up detection of salient events and coordinating appropriate responses and its activity is correlated with improved sound detection thresholds, showing a role in the direction of attentional resources toward audition. Main encephalographic findings linked to the salience network in patients with tinnitus report: (1) increased delta and gamma activity in the right anterior insula [72], (2) decreased theta and gamma activities in the left anterior insula [72], and (3) increased alpha activity in both the left and the right anterior insula [58]. The activation of the salience network in tinnitus patients suggests that the brain allocates an importance to the auditory stimulus and might as such also signify importance to the internally generated tinnitus sound. In addition, the insula cortex has distinct auditory and multisensorial connections (with the prefrontal and auditory cortices, amygdala, thalamus, parabrachial nucleus, orbitofrontal cortex, striate, cuneus, and cerebellum) that have been identified through functional imaging techniques to be dysfunctional in cases of severe tinnitus [88].

### 3.2. Tinnitus-Related Brain Structures “beyond” Auditory Pathways (Table 3)

Auditory cortex is connected to several other brain areas through extralemniscal auditory pathway elements such as limbic structures and through temporal lobe efferences [77]. The involvement of these areas seems to be concomitant to auditory structures dysfunctions and not exclusive of tinnitus pathogenesis.

fMRI studies show a complex involvement of multiple areas in tinnitus patients in comparison to healthy controls: auditory resting-state network has been found to encompass bilateral primary and associative auditory cortices, insula, prefrontal, sensorimotor areas, the brainstem, thalamus, NAc, isthmus of cingulate gyrus, right and left occipital, parietal, and PFC; in chronic tinnitus patients, as compared to controls, increased connectivity was found in the brainstem, cerebellum, right basal ganglia/NAc, parahippocampal areas, right frontal and parietal areas, left sensorimotor areas, and left superior temporal region. In addition, chronic tinnitus patients as compared to controls showed decreased connectivity in right primary auditory cortex, left fusiform gyrus, and left frontal and bilateral occipital regions [42]. Concomitant nucleus accumbens and primary auditory cortex hyperactivity associated with increased gray matter and decreased white matter concentrations in the ventromedial PFC were also found in a recent study of Leaver et al. [68].

Several MRI studies evidenced structural alterations in tinnitus patients involving grey matter decrease in auditory and nonauditory brain areas [67].

Diffusion tensor imaging (DTI) is an in vivo imaging tool for studying CNS microstructure [44]. That is, whereas conventional structural MRI is relatively insensitive to the white matter microstructure, DTI reveals the orientation of the white matter tracts in vivo and yields an index of microstructural integrity through quantification of the directionality of water diffusion [69]. Lee et al. used DTI to compare tinnitus subjects with control populations [89]: a statistically significant reduction in the fractional anisotropy (FA) value was found in frontal and parietal arcuate fasciculus in the tinnitus groups compared with the healthy control group. Another recent study by Benson et al. [70] showed increased FA in the inferior frontooccipital fasciculus and superior longitudinal fasciculus and decreased FA in the superior longitudinal fasciculus of the left parietal lobe. The arcuate fasciculus is a white-matter fibre tract, part of the superior longitudinal fasciculus, that links lateral temporal cortex with frontal cortex via a dorsal projection that arches around the Sylvain fissure; it connects Broca's area and Wernicke's area, playing a critical role in language functions. Other authors also confirmed the findings about “disconnectivity” in extra-auditory pathways involving PFC, temporal lobe, thalamus, and limbic system [71] in DTI studies. On the other hand, some authors described a right sided connectivity increase between the anterior cingulate and the frontal cortex and parietal cortex [76].

Among tinnitus patients there is a large heterogeneity of findings about functionality of brain structures and a positron emission tomography (PET) study evidenced gender-related differences in female tinnitus patients increased metabolic activity of left primary cortex was associated with a similar finding in temporal and parietal brain areas while in male patients an increased metabolic activity was found in frontal and occipital regions [53]. A concomitant involvement of right temporal and left frontal areas (marked reduction in alpha (8–12 Hz) together with an enhancement in delta (1.5–4 Hz) neuronal activity) was also reported in a study utilizing MEG [71].

Recently also dorsolateral prefrontal cortex (DLPFC) dysfunctions have been associated with tinnitus and tinnitus-related distress [53]. DLPFC exerts early inhibitory modulation of input to primary auditory cortex in humans [90] and has been found to be associated with auditory attention [91] resulting in top-down modulation of auditory processing [92]. As electrophysiological data indicated that tinnitus might occur as the result of a dysfunction in the top-down inhibitory processes [73], it has been hypothesized that the hypofunctioning of DLPFC may contribute to the hyperfunctioning of auditory cortex observed in tinnitus patients, representing a neurophysiological substrate of tinnitus perception and related distress [69]. An electroencephalographic (EEG) study recently confirmed the involvement of DLPFC (associated with a less synchronized alpha activity in the posterior cingulate cortex and precuneus and with a concomitant more synchronized alpha activity in subcallosal anterior cingulate cortex, the insula, parahippocampal area, and amygdala) in tinnitus distressed patients [72].

A tinnitus distress MEG study, in addition, associated tinnitus with an increased right sided connectivity between the anterior cingulate and the frontal cortex and parietal cortex [76].

## 4. Discussion

Far from being considered only an otological disorder, tinnitus is a frequent and heterogeneous symptom of various underlying pathologies, resulting in most cases from neuronal changes occurring in the CNS as a reaction to auditory deprivation. As tinnitus-related plastic rearrangements of auditory pathways involve brain structures such as insula, IC, thalamus, and PFC that are important nodes of various other brain circuits, it can be hypothesized that these rearrangements lead not exclusively to auditory symptoms but also to other symptomatology involving psychic functions.

Results from neuroimaging (MRI, fMRI, and PET) and encephalographic (MEG and EEG) studies widely documented tinnitus-related processes of neural plasticity that affect neuronal activity of the auditory system at several levels along the auditory pathway as well as cortical regions involved in perceptual, emotional, memory, attentional, and salience functions [93]. Among the alterations observed in tinnitus, some altered networks are also involved and play a critical role in the physiopathology of emotional and psychiatric disturbances, supporting the idea of overlapping neurobiological substrates between decompensated tinnitus and psychopathology.

Consistently with the presented results, tinnitus, initially related to a clinicoanatomical approach based on a narrow cortical localizationism within an otological perspective, could be better explained by an holistic approach [94] considering all regions to be mutually interconnected through a network of homogeneously distributed association fibres or by associationist models considering the brain organized in parallel distributed networks around cortical epicentres [95].

Considering that psychological functions and symptoms are the result of the simultaneous activity of all brain regions acting as a whole through association pathways, psychic functions and tinnitus may be considered emergent properties of partially overlapping large-scale neural networks [96, 97].

The discussion section will be presented in two separate sections each section discussing those brain networks that could underlie the still not adequately understood connection between tinnitus and psychopathology.

### 4.1. Tinnitus-Related Brain Structures “into” and “within” Auditory Pathways

The hyperactivity of auditory cortex plays a critical role both in tinnitus and in auditory verbal hallucinations (AVHs); this evidence is supported by the fact that inhibitory temporal transcranial magnetic stimulation (TMS) protocols have successfully been used to treat both of the disorders [98]. For what concerns AVHs, defined as “the subjective experience of hearing voices speaking in the absence of corresponding physical stimulation,” it has been proposed that the brain regions dedicated to auditory processing, especially the primary auditory cortex, are relevant to experiencing hallucinations. This idea is supported by the so-called “symptom capture” studies, which attempt to measure brain activity while subjects are experiencing AVHs [99–101].

Even if in the majority of cases relevant clinical differences between tinnitus and AVHs are present, both the clinical conditions may be considered forms of auditory perception alterations which present with a “continuum of complexity” and with subjective differences in the levels of insight and perceived distress, having potential similar neurobiological substrates [102].

Tinnitus differs from AVHs because it is perceived as a sound not having any complex, digitalized linguistic meaning, thus being typically recognized by patients as a pathological phenomenon. There is evidence suggesting that, while tinnitus and AVHs share common dysfunctions in auditory processing underlying phantom sound perceptions, they present a different pattern of alterations of thalamocortical networks that are supposed to be related to conscious perception of auditory inputs [52, 102].

Behrendt [103] has provided a thought-provoking hypothesis based on the idea that perceptual experience arises from synchronization of gamma oscillations. This oscillatory activity is normally constrained by sensory input and also by prefrontal and limbic attentional mechanisms. There is evidence that in patients with schizophrenia (SCZ) there is impaired modulation of thalamocortical gamma activity by external sensory input, allowing attentional mechanisms to play a preponderant role in the absence of sensory input and thus potentially leading to hallucinations. While dysfunctions of auditory cortex are related to AVHs perception, functional alterations of extralemniscal auditory pathways structures represent a common field between tinnitus and other psychopathological dimensions. In fact, direct connections from the thalamic nuclei of the nonlemniscal pathway to the amygdala, the hippocampus, and other structures of the limbic system may explain, according to several authors, the affective components of tinnitus [34].

Limbic dysfunction underlies many symptoms (related to emotion regulation and social interaction and behaviour) of psychiatric conditions, including SCZ, affective disorders, psychopathy, and autism spectrum disorders (ASD) [83]. This system has often been considered a “switch” in the brain that can turn the tinnitus sensation on or off [94]. The first behavioral animal model of tinnitus developed by Jastreboff et al. in 1988 [104] has provided important insight into the neuronal mechanisms involved in the pathophysiology of tinnitus; it does not exist, however, an animal model of tinnitus-related distress potentially representing the psychopathological consequences of tinnitus. Increased activity in the auditory cortex as a consequence of auditory deprivation, in fact, is necessary but not sufficient for tinnitus perception: the patient becomes distressed by the phantom sound if auditory activity is connected to larger coactivated networks involving, also, the limbic system [105, 106]; related psychiatric symptoms could derive from dysfunction of circuits of the limbic system, not directly from topological structures [83].

The limbic structures that are known to be related to tinnitus pathophysiology (amygdala, hippocampus, parahippocampal gyrus, insula, cingulum, and, for extension, nucleus accumbens) are components of three distinct but partially overlapping networks and corresponding clinical syndromes [83]. The first network, composed of the hippocampal-diencephalic limbic circuit (connected through the fornix and mammillothalamic tract) and the parahippocampal-retrosplenial circuit (ventral cingulum), is dedicated to memory and spatial orientation, respectively; the second, the temporoamygdala-orbitofrontal network (connected through the uncinate fasciculus) is dedicated to the integration of visceral and emotional states with cognition and behavior; the third, the dorsomedial default-mode network consists of a group of medial regions (anterior cingulate-medial PFC and the posterior cingulate-precuneus interconnected through the dorsal cingulum). Psychiatric disorders associated with these networks are described in Table 4.

Tinnitus distress seems to be also related to neural activity in the left and right anterior insula. Insular cortex through interconnection with cingulate gyrus, orbitofrontal cortex, and parahippocampal gyrus (paralimbic areas) is believed to be involved in consciousness and plays a role in diverse functions including perception, motor control, self-awareness, social cognition, cognitive functioning, and interpersonal experience [107]. As written above, the insula together with the dorsal anterior cingulate cortex has also been referred to as the salience network [87]; the activation of the salience network in tinnitus patients suggests that the brain allocates high importance to the internally generated tinnitus sound. Anomalies of salience network have been implicated in different psychiatric disorders, especially SCZ [108], ASD, and attention-deficit hyperactivity disorder (ADHD) [109], as well as obsessive compulsive disorder (OCD) [110], anxiety, and mood disorders [111]. These clinical conditions (SCZ and ASD in particular) are characterized by difficulties in integrating external sensory stimuli with internal states, and several authors postulated the key role of aberrant salience in their physiopathology [112]. The paralimbic involvement in tinnitus patients may thus indicate tinnitus distress as a state of aberrant salience potentially comparable to the aberrant salience of other serious brain disorders.

Tinnitus usually becomes troublesome if patients focus their attention on it and the perception of tinnitus severity usually correlates more closely with psychological and general health (such as pain or insomnia) factors than with audiometric parameters [105]. The perception of tinnitus often extinguishes in a short time through habituation mechanisms: superior brain centres activate thalamic filters to “switch off” the signal, often independently of the resolution of the dysfunction that originally generated the tinnitus. On the other hand, in case of emotional reinforcements caused by fear, anxiety, or tension, the continued perception of tinnitus is supported by the limbic system, primarily by the amygdala; this establishes a vicious circuit which leads to the amplification (increased excitability) and the chronicity (through neuronal plasticity mechanisms) of the signal [10].

From a clinical point of view, emotional “limbic” reinforcements can strongly worsen the tinnitus-related distress potentially representing the milestones to shift from a compensated to a decompensated tinnitus [113]. Consistently, pharmacological (selective serotonin reuptake inhibitors [114, 115], benzodiazepines [116], mood stabilizers [117, 118]), psychotherapeutic (cognitive behavioural therapy [119]) and neuromodulating (TMS [120, 121], tDCS [122], Neurofeedback [123]) treatments aimed at modulating the subjective emotional component of tinnitus showed to be among the best interventions to treat tinnitus distress and should always be integrated with regular otological interventions [10].

### 4.2. Tinnitus-Related Brain Structures “beyond” Auditory Pathways

Among the brain areas beyond auditory cortex, the frontal lobe seems to be the principal structure involved in the pathogenesis of tinnitus. The role of frontal lobe in tinnitus has been confirmed by studies using different brain mapping techniques and it involves frontocortical and frontosubcortical circuits.

Data from DTI studies in tinnitus patients show decreased fractional anisotropy in frontal and parietal arcuate fasciculus [69], increased FA in the inferior frontooccipital fasciculus and superior longitudinal fasciculus, decreased FA in the superior longitudinal fasciculus of the left parietal lobe [89], “disconnectivity” in extra-auditory pathways involving pathways involving PFC, temporal lobe, thalamus, and limbic system [71] and increased right sided connectivity between anterior cingulate, frontal and parietal cortices [76].

Of particular interest are data on the arcuate fasciculus, this pathway is critically involved with human language. Evidence of arcuate fasciculus damages in patients with tinnitus indicates a deterioration of white-matter fibres and underlines the importance of cortical interconnectivity in the pathogenesis of this disorder. Arcuate fasciculus has also been found damaged in several psychiatric disorders such as ASD, SCZ, dyslexia, and dyscalculia, supporting the idea that white matter deterioration may represent a common functional substrate of tinnitus and psychiatric disorders. Moreover, as Tim Crow assessed in the paper “Schizophrenia as the price that homo sapiens pays for language: a resolution of the central paradox in the origin of the species” [124] there is a well-established involvement of language development in psychiatric disorders, supporting the idea of a potential role of arcuate fasciculus damages in both the conditions.

The concomitant involvement of right temporal and left frontal areas (marked reduction in alpha (8–12 Hz) together with an enhancement in delta (1.5–4 Hz) neuronal activity) reported in a MEG study [71] could derive from the interconnection of the two lobes through the arcuate fasciculus. There is also evidence of the involvement of other long white tract fibres pathways in tinnitus and psychopathology: an altered network among frontooccipital connections [36, 53, 78] has been associated with behavioural syndromes like personality changes, emotional liability, and disinhibition [107]; lesion at the longitudinal superior fasciculus leading to an altered connectivity between frontal cortex, cingulus, and parietal cortex [76] has been hypothesized to determine derealization symptomatology and memory deficits [125]; OCD symptomatology has been suggested to be related to a dysfunction of frontoparietal connectivity [126].

Alterations in frontal-subcortical circuits [71] from PFC to thalamus and limbic system seem to be relevant for the onset of several psychiatric disorders such as depression, OCD, and SCZ [127]. Among frontal-subcortical circuits, DLPFC exerts early inhibitory modulation of input to primary auditory cortex in humans and several studies evidenced its involvement in tinnitus; as electrophysiological data indicated that tinnitus might occur as the result of a dysfunction in the top-down inhibitory processes [73], it has been hypothesized that the hypofunctioning of DLPFC may contribute to the hyperfunctioning of auditory cortex observed in tinnitus patients, representing a neurophysiological substrate of tinnitus [69]. Results from a large body of functional and structural brain imaging studies provide convergent evidence that DLPFC plays critical roles in mood regulation and DLPCF hypoactivity is nowadays considered a critical neural substrate for depression [128]. Impaired DLPFC functioning may thus represent a common neurobiological substrate of tinnitus symptomatology and depression, potentially explaining the high rate of comorbidity between the two disorders and the efficacy of prefrontal TMS in the treatment of both of the disorders [29, 103, 129, 130]. Consistently with this view, Gray described PFC as a “candidate for the integration of sensory and emotional aspects of tinnitus” [131].

Furthermore, concomitant hyperactivation of NAc and primary auditory cortex and decreased white matter concentrations in the ventromedial PFC [68] have been proposed as indirect findings related to frontosubcortical circuits involvement in patients with tinnitus. NAc is involved in both normal and abnormal reward processes, in the pathogenesis of anhedonia and loss of motivation. Due to its strategic location between emotional system, cognitive system, and motor control system, NAc has been proposed as a central node in mood and feeling regulation [132].

Finally, implication of extra encephalic structures as cerebellum in a circuit involving brainstem, basal ganglia/NAc, parahippocampal, right prefrontal, parietal, and sensorimotor areas [42] should be related to psychiatric manifestation (SCZ, bipolar disorder, major depressive disorder, anxiety disorders, dementia, and ADHD) [133, 134].

## 5. Conclusion

From an accurate analysis of scientific literature it emerges that tinnitus and psychiatric disorders share common neuronal network dysfunctions related to specific pathways; thalamus and limbic areas seem to represent the most relevant “nodes” of such altered networks linked to auditory extralemniscal areas, while multiple hodological alterations of frontal circuits with others structures seem to emerge from extra-auditory involvements in tinnitus. The rearrangement of auditory cortex functionality is probably linked to tinnitus perception.

From a tractographic point of view, it is possible to hypothesize that neuroplastic rearrangements of auditory pathways in patients with tinnitus could affect the functionality of all those nonauditory brain areas connected with the auditory cortex through the plastic rearrangement of white matter pathways potentially leading to the onset of psychopathological symptoms. On the other hand it is possible that psychological stress, current or previous psychiatric disorders, and personality traits associated with a genetically or epigenetically determined vulnerability may represent a vulnerability factor giving rise to maladaptive tinnitus-related neuroplastic rearrangements [135] leading to tinnitus symptomatology. Given the above, our hypothesis is that patients' symptomatology may be considered the peculiar expression of an alteration of global brain hodological equilibrium.

Clinical trials concerning the use of psychotropic medications for the treatment of tinnitus evidence interesting issues supporting this hypothesis: standard tinnitus treatments often show poor outcomes on tinnitus-related distress [136–138] while the treatments focused on psychiatric comorbidities appear to be more effective than standard tinnitus treatments, achieving a response rate of up to 81.39% [139].

Among psychiatric treatments, the best outcomes have been obtained approaching psychopathological disturbances with a dimensional rather than a DSM-defined categorical point of view [139]; the categorical model of the DSM, in fact, provides a poor fit to the latent structure of psychopathology [140]. Dimensional approaches to psychiatric therapies have increasingly been supported; to this purpose, Buckholtz and Meyer-Lindenberg have recently proposed a dimensional transdiagnostic “common symptom, common circuit” model of psychopathology suggesting that specific clusters of psychic disturbances correspond to specific clusters of brain network alterations associated with tinnitus perception [63, 141]. We find reliability and promise in this kind of approach in order to diagnose and treat psychiatric comorbidities of tinnitus.

The hodological view of psychiatric comorbidities in tinnitus patients also gives rise to other considerations: (1) “$o\delta \overset{´}{\text{o}}\varsigma$” means “way,” but also “connection”: the management of tinnitus complexity requires a multidisciplinary approach where otolaryngologists should involve and “connect” several different medical specialists; (2) clinicians should have more accurate instruments to assess the psychiatric comorbidities and the global neurofunctional activity [11]; (3) other nonpsychiatric comorbid conditions potentially able to induce plastic rearrangements, such as muscle tension [4] and hyperinsulinemia [142, 143], should be taken into consideration; (4) from a “*methodological*” point of view, the studies on tinnitus pathogenesis and on treatment response should be personalized rather than standardized.

Given the absence of objective diagnostic markers, tailored psychiatric treatments can currently be implemented exclusively on the basis of patients' reported complaints [144–146]. We hereby suggest a comprehensive approach to tinnitus treatment focused on 4 areas of intervention based on its clinical presentations: (A) predominantly audiological (deafferentation or deprivation tinnitus); (B) predominantly somatosensory (i.e., cross-modal tinnitus); (C) predominantly psychopathological (D) mixed-combined [10]. Further studies are needed to evaluate specific therapeutic approaches targeted on each of these 4 clinical domains. It is also probable that if functional and structural imaging studies will follow an adequate classification of tinnitus patients they will be able to provide more detailed and less confusing results.

## Figures and Tables

**Table 1:** Common systemic neurootological risk factors for developing tinnitus [36].

Otological, infectious	Otitis media, labyrinthitis, mastoiditis
Otological, neoplastic	Vestibular schwannoma, meningioma
Otological, labyrinthine	Sensorineural hearing loss, Ménière's disease, vestibular vertigo
Otological, other	Impacted cerumen, otosclerosis, presbycusis, noise exposure
Neurological	Meningitis, migraine, multiple sclerosis, epilepsy
Traumatic	Head or neck injury, loss of consciousness
Otofacial	Temporomandibular joint disorder
Cardiovascular	Hypertension
Rheumatological	Rheumatoid arthritis
Immune-mediated	Systemic lupus erythematous, systemic sclerosis
Endocrine and metabolic	Diabetes mellitus, hyperinsulinaemia, hypothyroidism, hormonal changes during pregnancy
Ototoxic medications	Analgesics, antibiotics. Antineoplastic drugs, corticosteroids, diuretics, immunosuppressive drugs, nonsteroidal anti-inflammatory drugs, steroidal anti-inflammatory drugs

**Table 2:** Tinnitus-related alterations “into” and “within” auditory pathways.

Methods	Alterations observed	References
MRI	Reduced grey matter volume in bilateral auditory areas including the Heschl's gyrus.	[43]
	Significant grey matter decrease in the right IC.	[44]

fMRI	Abnormal asymmetric IC activation in patients with lateralized tinnitus.	[45]
	The ratio of activation between right and left IC did not differ significantly between tinnitus and non-tinnitus patients or in a manner dependent on tinnitus laterality.	[46]
	Tinnitus-induced hyperactivity in the dorsal cochlear nucleus.	[47]
	Tinnitus-related hyperexcitability of auditory cortex.	[48]
	Significant signal change lateralized towards the side of perceived tinnitus in primary auditory cortex and IC in patients with right sided tinnitus and towards the medial geniculate body in patients with left sided tinnitus.	[49]
	Smaller medial partition of Heschl's gyrus gray matter volume.	[50]

PET	Tinnitus-related elevated blood flow in auditory cortex.	[51]
	Focal metabolic activation in the predominant left auditory cortex.	[52]
	Significantly increased metabolic activity in the left primary auditory cortex; increased metabolic activity in temporal and parietal brain regions (in female tinnitus patients) and in frontal and occipital regions (in male tinnitus patients).	[53]
	Asymmetric activation of the auditory cortex, predominantly on the left side and independently from tinnitus laterality.	[54]
	Activation of left and right posterior inferior temporal gyrus as well as left and right posterior parahippocampal-hippocampal interface; overactivation of left in contrast to right Heschl's gyrus independently from tinnitus laterality.	[55]

MEG	Reduced alpha activity (8–12 Hz) and increased slow wave activity (delta and theta 1–6 Hz) and gamma activity (>30 Hz) in the temporal cortex.	[56]

EEG	Abnormal gamma band activity (>30 Hz) generated as a consequence of hyperpolarization of specific thalamic nuclei.	[57]
	Correlation between electroencephalographic gamma band activity in the contralateral auditory cortex and the presence of tinnitus.	[58]
	Discrete localised unilateral foci of high frequency activity in the gamma range (>40–80 Hz) over the auditory cortex.	[59]
	Reduced wave I (indicating reduced auditory-nerve activity) and elevated waves III and V amplitude (indicating hyperactivity of pathways originating from ventral cochlear nucleus) assessed via auditory brainstem responses.	[60]
	Increased neuronal activity in auditory pathways (long latency auditory evoked potentials).	[61]
	Cortical information processing dysfunction in chronic tinnitus patients associated with auditory stimuli.	[62]

IC: Inferior Colliculus.

**Table 3:** Tinnitus-related alterations “beyond” auditory pathways.

Methods	Alterations observed	References
fMRI	Increased connectivity in extra-auditory regions (brainstem, basal ganglia/NAc, cerebellum, parahippocampal, and right prefrontal, parietal, and sensorimotor areas); reduced connectivity in right primary auditory cortex, left prefrontal, left fusiform gyrus, and bilateral occipital regions.	[42]
	Reduced grey matter volume in bilateral insula.	[43]
	Significant grey matter decrease in right IC and left hippocampus.	[44]
	Hyperactivity in the anterior cingulate cortex, midcingulate cortex, posterior cingulate cortex, left middle frontal gyrus, retrosplenial cortex and insula.	[65]
	Highly significant volume loss in the subcallosal area; significant increase of grey-matter density in the posterior thalamus.	[66]
	Activation of primary auditory cortices, associative auditory cortices, and left hippocampus.	[67]

PET	Hyperactivity of NAc and primary auditory cortex; increased gray matter and decreased white matter concentrations in the ventromedial PFC.	[68]
	Increased metabolic activity in temporal and parietal brain regions (in female tinnitus patients) and in frontal and occipital regions (in male tinnitus patients) associated with significantly increased metabolic activity in the left primary auditory cortex.	[53]

DTI	Decreased FA in the left frontal arcuate fasciculus and the right parietal arcuate fasciculus.	[69]
	Increased FA in the inferior frontooccipital fasciculus and superior longitudinal fasciculus; decreased FA in the superior longitudinal fasciculus of the left parietal lobe.	[70]
	Disrupted white matter integrity in tracts involving the connectivity of PFC, temporal lobe, thalamus, and limbic system.	[71]

EEG	Increased alpha activity in both left and right anterior insula in patients with severe tinnitus-related distress who can or cannot cope with these phantom sounds.	[72]
	In the right anterior insula increased delta and gamma activity related to increased tinnitus distress; in the left anterior insula decreased theta and gamma activities.	[58]
	Gamma-band activity in the parahippocampal area contralateral to the tinnitus lateralization.	[72]

MEG	Marked reduction in alpha (8–12 Hz) power associated with enhancement in delta (1.5–4 Hz) neuronal activity particularly in right temporal and left frontal areas	[56]
	In patients with significant tinnitus-related distress, more synchronized alpha activity in subcallosal anterior cingulate cortex, insula, parahippocampal area, and amygdala; less synchronized alpha activity in posterior cingulate cortex, precuneus, and DLPFC.	[58]
	Tinnitus-related distress correlated with a right sided connectivity increase between the anterior cingulate and the frontal and parietal cortices.	[46]
	Altered role of frontal cortex in the modulation of sensory inputs.	[73]

IC: Inferior Colliculus

NAc: Nucleus Accumbens

PFC: Prefrontal Cortex

DLPFC: Dorsolateral Prefrontal Cortex

FA: Fractional Anisotropy.

**Table 4:** Limbic networks and neuropsychiatric disorders [65].

Network	Disorder
*Hippocampal-diencephalic and parahippocampal-retrosplenial *	(i) Amnesias (ii) Korsakoff's syndrome (iii) Mild cognitive impairment (iv) Alzheimer's disease (early) (v) Balint syndrome

*Temporoamygdala-orbitofrontal *	(i) Alzheimer's disease (advanced) (ii) Semantic dementia (iii) Kluver-Bucy syndrome (iv) Temporal lobe epilepsy (v) Geschwind's syndrome (vi) Psychopathy (vii) Bipolar affective disorders

*Dorsomedial default network *	(i) Depression (ii) Autism (iii) Schizophrenia (iv) Obsessive compulsive disorder (v) Mild cognitive impairment (vi) Alzheimer's disease (early) (vii) Attention deficit hyperactivity disorder (viii) Anxiety
